# Health Costs and Quality of Life Among Diabetic Foot Ulcer Patients in South India: A Cross-Sectional Study

**DOI:** 10.7759/cureus.79950

**Published:** 2025-03-03

**Authors:** Trupti Bodhare, Samir Bele, Balakumaran B, Ramji M, Gavin Francis J, Shalini V, Thendral Muthiah, Bharath Rajh

**Affiliations:** 1 Community and Family Medicine, All India Institute of Medical Sciences, Madurai, Madurai, IND; 2 Community Medicine, Velammal Medical College Hospital & Research Institute, Madurai, IND

**Keywords:** catastrophic health expenditures, diabetic foot ulcer, health-related quality of life, out-of-pocket expenditure, patients

## Abstract

Introduction: The financial burden of managing diabetic foot ulcers (DFUs) can lead to catastrophic health expenditure (CHE) and financial hardship, ultimately impacting the quality of life (QoL) of patients. The relationship between healthcare costs and health-related quality of life (HRQoL) is critical for understanding the broader implications on patients' physical, psychological, and social well-being.

Methodology: A cross-sectional study was conducted among DFU patients selected using convenient sampling. A semi-structured questionnaire was used to assess patients' socio-demographic and clinical characteristics. HRQoL was assessed using the 36-Item Short Form Survey (SF-36).

Results: The sample population was 173 patients, including 125 (72.3%) male and 48 (27.7%) female patients. The mean age was 57.80 ± 10.51 years. The total out-of-pocket expenditure (OOPE) amounted to Rs. 89317.51 ± 150378.81 ($1134.33 ± 1909.81). Around 81 (46.8%) patients were experiencing CHE. The socioeconomic status, duration of diabetes, and severity of wounds significantly influence the likelihood of experiencing CHE (p < 0.05). Lower QoL scores were obtained in the domain of physical functioning (37.23 ± 23.26), role physical (17.34 ± 30.55), and role emotional (18.11 ± 32.64). Gender, occupational status, comorbidities, wound duration, Wagner's grading of ulcers, and CHE significantly impact various domains of HRQoL (p < 0.05).

Conclusion: The substantial healthcare costs of managing DFUs can result in financial strain, negatively influencing patients' QoL. Therefore, it is essential to focus on both the financial implications of diabetes management and the enhancement of patients' QoL to achieve better health outcomes.

## Introduction

Diabetes is a major public health concern with far-reaching consequences for the community's physical, psychological, and economic well-being. The International Diabetes Federation (IDF) estimates that the number of people with diabetes, currently at 537 million, will rise to 783 million by 2045. With more than three-quarters of individuals with the disease living in low- and middle-income nations, 6.7 million fatalities were attributed to diabetes in 2021 [1,2]. From 1990 to 2016, there was a significant increase in disability-adjusted life years (DALYs) associated with diabetes [3]. Diabetes led to a staggering US$ 966 billion in healthcare costs, marking a significant 316% surge in just 15 years, and is expected to rise to US$ 1054 billion by 2045 [2].

India accounts for one in every seven people with diabetes globally. The National Family Health Survey (NFHS-5) reported a diabetes prevalence of 16.1%. With an estimated one million diabetes-related deaths annually, the number of diabetics is predicted to increase from 74.2 million to 124.9 million by 2045 [4]. According to the IDF tenth edition statistics, India's total spending on diabetes was US$ 8485.8 million, with an average expenditure of US$ 114.4 per person [2].

Diabetic foot ulcer (DFU) is a severe pathophysiological consequence often caused by poor glycemic control, neuropathy, peripheral vascular disease, or improper foot care. It is a prevalent cause of foot osteomyelitis, lower extremity amputation, and increased mortality. There is a significant disparity in the mortality rate between individuals with DFU (231 deaths per 1000 person-years) and those with diabetes but without foot ulcers (182 deaths per 1000 person-years), with people belonging to lower socioeconomic status having a higher incidence of DFU and consequent amputation. Annually, 18.6 million individuals get DFU globally. The findings of a systematic review and meta-analysis indicate that the prevalence of DFU worldwide is 6.3% [4]. The prevalence of DFUs is increasing [5,6], particularly in both developing and underdeveloped countries, with an estimated 15-25% of diabetes patients in India expected to develop DFU [7,8].

Lack of information, inadequate health infrastructure, and economic constraints may worsen this problem. DFUs in India are a significant cause of hospital admissions, non-traumatic amputations, and increased mortality. The cost of DFU care in India is significantly high. The research results indicate that persons with DFU incur four times greater expenses than those without DFU. Furthermore, it takes an average patient about 68.8 months of their salary to pay the costs associated with complete DFU care [9,10]. A recent study in Chennai indicated that the median annual out-of-pocket expenditure (OOPE) for DFU therapy was Rs. 29,775 (Rs. 9650-81,120) [11].

Healthcare providers frequently use health-related quality of life (HRQoL) measures as important performance indicators, expecting them to demonstrate progress in improving HRQoL as part of their treatment efforts, particularly when providing value-based services. Furthermore, the assessment of HRQoL provides an opportunity to address the unique physical or mental health concerns of patients [12]. Research has shown that DFUs can have significant physical, psychological, and social consequences. It results in decreased social engagement, increased strain on patients and their caregivers, limited work opportunities, and financial difficulties. Patients experience a decline in their HRQoL due to increased hospital admission rates and the high cost of treating DFUs. Increased medical expenses can lead to financial difficulties, which can negatively affect both the quality of life (QoL) and clinical state. It is also noteworthy that financial hardship brought on by medical expenditure could adversely affect the QoL along with clinical status [11,13].

Healthcare expenditure estimates, including the proportion of catastrophic health expenditure (CHE), can assist policymakers in allocating resources and developing a comprehensive strategy for cost control and integrated health coverage. This knowledge would also help develop cost-effective DFU models. Furthermore, it would be beneficial to gain a deeper comprehension of the relationship between expenses and HRQoL, as well as to compare these data with other regions.

Until now, there have been only a few studies that have systematically investigated the financial burden and its impact on the QoL for individuals with DFU in India. This study was conducted to determine the association between CHE and HRQoL among patients with DFU in a South Indian population. The researchers sought to analyze the impact of healthcare costs on various factors and domains of QoL of these patients.

## Materials and methods

Study design and setting

This was a hospital-based cross-sectional study. The study was carried out in a tertiary care teaching hospital in South India. The study was conducted from June 2022 to August 2023.

Participants

Participants included individuals with DFU who were admitted to the department of general surgery.

Inclusion and exclusion criteria

All patients with DFU aged 18 years or above who agreed to participate in the study and provided written informed consent were enrolled. Patients with moderate to severe cognitive impairment or who were unable to communicate owing to a serious morbid illness or disability were excluded from the research.

Variables and data measurements

The questionnaire was divided into two parts. Part A involved the use of a semi-structured questionnaire to assess demographic details, socioeconomic characteristics, and various risk factors for DFUs. We assessed the participants' socioeconomic status using a revised version of the modified Kuppuswamy socioeconomic scale using the Consumer Price Index (CPI) for June 2023. [14] The questionnaire covered important clinical aspects, including the duration of diabetes, glycemic status, comorbidities, and duration of treatment for diabetic ulcers. Details of the ulcer site, tenderness, and presence of purulent discharge, etc., were carefully documented. Ulcers were graded using Wagner's classification.

The explicit expenses for medical and non-medical purposes were documented. The direct medical cost per patient was evaluated by considering expenses for medical consultations, laboratory tests, antibiotic treatment and other medication expenses, hospital charges, including admission fees, surgical debridement, minor or major amputations, and revascularization procedures, etc. Non-medical costs include transportation costs, the cost of accompanying attendants, and the loss of wages/income for both the patient and attendants. OOPEs are the expenditures made by patients and their families from their financial resources (aside from insurance coverage) for healthcare services or items to treat DFUs. CHE was considered if the patient's household spending on DFU management exceeded 10% of total household expenditure or income [15].

We utilized the widely accepted HRQoL tool, the 36-Item Short Form Survey (SF-36), which is commonly regarded as the benchmark for evaluating patient-reported outcome measures. The SF-36 has strongly correlated with wound-specific outcome instruments, including the Diabetic Foot Ulcer Scale and the Cardiff Wound Impact Schedule [13]. It encompasses eight distinct health concepts: physical functioning (PF), role physical (RP), bodily pain (BP), general health (GH), energy/fatigue (EF), social functioning (SF), role emotional (RE), and emotional well-being (EW). In the SF-36 analysis, raw scores are translated into transformed scores on a 0-100 scale for eight subscales, with a higher score indicating a better health status or QoL.

Study size and sampling

The research comprised individuals with DFUs who were admitted to the general surgery ward for treatment, using convenient sampling. Based on the results of a previous study, the sample size for the patients was determined to be 173 using the formula: $n = \frac{z^2 p(1-p)}{d^2}$. With a 95% level of significance, a prevalence of DFU of 13%, and a 5% margin of error [16], the required sample size for the present study was 173.

Pilot study

A pilot study was done with a sample size of 30 participants to evaluate the appropriateness of the study instrument in terms of its technical feasibility and the accuracy of question presentation and sequencing. The pilot research participants were not included in the original study sample.

Ethical considerations

Prior to initiating the research, we obtained approval from the Institutional Ethics Committee, Velammal Medical College Hospital and Research Institute (VMCIEC/43/2021). The researchers have presented a thorough explanation of the study, emphasizing that participation is completely voluntary and that individuals can withdraw at any time without any negative repercussions. We obtained written informed consent from all study participants. With a focus on maintaining high levels of confidentiality, participant-identifying data were securely stored and replaced with code numbers. The data were secured with passwords to ensure confidentiality.

Statistical analysis

R programming version 4.4.1 (R Foundation for Statistical Computing, Vienna, Austria) was used for the statistical analysis. The analysis encompassed descriptive statistics such as frequencies, percentages, means, standard deviations, medians, and interquartile ranges to summarize the socio-demographic and clinical characteristics of the study population. The costs are expressed in both Indian rupees and US dollars based on the yearly average exchange rate (2022). A logistic regression analysis was performed to identify key predictors of CHE. The study employed multivariate analysis of variance (MANOVA) to analyze the impact of a variety of risk factors on distinct domains of HRQoL. Significance was attributed to p-values below 0.05. The statistical analysis included binary logistic regression, where coefficients (β) and 95% confidence intervals (CI) were reported to identify key predictors of CHE. For MANOVA, the F-values and corresponding p-values were provided to assess the combined effect of risk factors on different HRQoL domains. Assumptions for both analyses were tested to ensure model validity.

## Results

Table 1 describes the sample population, which consisted of 173 patients, including 125 males (72.3%) and 48 females (27.7%). The mean age was 57.80 ± 10.51 years. Most patients (132, 76.3%) were married, and the family type was predominantly nuclear (122, 70.5%). Around 120 (69.4%) patients had completed their schooling, while 132 (76.3%) were employed. The median family income was Rs. 216,000 (95% CI: 211,336.52-488,132.28) ($2,743.20 ($2,683.98-$6,199.28)), with 107 (61.8%) belonging to the middle-class category and 66 (38.2%) belonging to the lower-class category. In addition, 36 (20.8%) of the individuals were smokers, 10 (5.8%) used tobacco products, and 28.3% of the respondents said that they consumed alcohol.

**Table 1:** Socio-demographic characteristics of diabetic foot ulcer patients.

Variables	Frequency (n)	Percentage (%)
Gender		
Male	125	72.3
Female	48	27.7
Age in years	57.80 ± 10.51 (mean ± SD)	
Marital status		
Married	132	76.3
Unmarried	2	1.2
Widowed	39	22.5
Type of family		
Nuclear	122	70.5
Joint	34	19.7
Three generations	17	9.8
Occupational status		
Employed	132	76.3
Unemployed	41	23.7
Educational status		
Illiterate	27	15.6
Schooling	120	69.4
Diploma	17	9.8
Graduate	9	5.2
Family income in Rs. (₹)	216000 (211336.52–488132.28, 95% CI)	
Socio-economic status		
Lower class	1	0.6
Upper lower	65	37.6
Lower middle	54	31.2
Upper middle	53	30.6
Smoking		
Yes	36	20.8
No	137	79.2
Tobacco products		
Yes	10	5.8
No	163	94.2
Alcohol		
Yes	49	28.3
No	124	71.7
Health insurance		
Enrolled	21	12.1
Not enrolled	152	87.9

Table 2 describes the clinical features of DFU patients. It revealed that the average duration of diabetes among the participants was 11.87 ± 9.17 years. The median treatment period for diabetic ulcers was 30 days (IQR: 11.50-68.50 days). The average random blood sugar level was 229.18 mg/dl ± 84.18 mg/dl. The mean glycosylated hemoglobin (HbA1c) level was 8.11 ± 1.92. Approximately 95 (55%) wounds showed purulent discharge, and 116 (67.1%) reported tenderness. The majority of ulcers (58/33.5%) were classified as a deeper, full-thickness extension (Wagner's grade II), followed by grade III (47, 27.2%) and grade IV (40, 23.1%).

**Table 2:** Clinical characteristics of diabetic foot ulcer patients. HbA1c: glycosylated hemoglobin.

Variables	Frequency (percentage)
Duration of diabetes (mean ±SD)	11.87 ± 9.17
Random blood sugar (mg/dl) (mean ±SD)	229.18 ± 84.18
HbA1c (%) (mean ±SD)	8.11 ± 1.92
Duration of treatment for diabetic ulcer in days (median IQR)	30 (11.50–68.50)
Site of wound	
Lateral aspect	7 (4)
Medial aspect	10 (5.8)
Sole	58 (33.5)
Toe involvement	35 (20.2)
Dorsum of the foot	53 (30.6)
Others	10 (5.8)
Duration of ulcer	
<1 month	68 (39.3)
1-3 months	68 (39.2)
>3 months	37 (21.4)
Purulent discharge from wound	
Yes	95 (54.9)
No	78 (45.1)
Tenderness	
Yes	116 (67.1)
No	57 (32.9)
Wagner’s grading	
Superficial ulcer (I)	17 (9.8)
Deeper, full-thickness extension (II)	58 (33.5)
Deep abscess formation or osteomyelitis (III)	47 (27.2)
Partial gangrene of forefoot (IV)	40 (23.1)
Extensive gangrene(V)	11 (6.4)

Table 3 illustrates the OOPE associated with DFU in DFU patients. The total OOPE amounted to Rs. 89317.51 ± 150378.81 ($1135.34 ± 1909.81). The medical cost amounted to Rs. 72218.33 ± 145815.50 ($917.38 ± 1851.87), while the non-medical expenses totaled Rs. 17099.17 ± 16245.31 ($217.16 ± 206.32). The lowest mean value observed was for consultation costs, which was Rs. 36.43 ± 153.27 ($0.46 ± 1.95), and the highest was observed for costs due to hospitalization, which amounted to Rs. 41340.31 ± 80799.37 ($524.02 ± 1026.55). In addition, a significant portion of the patients (81, 46.8%) were experiencing CHE.

**Table 3:** Out-of-pocket expenditure for diabetic foot ulcers in diabetic foot ulcer patients.

S. No.	Variables	Percentage	Mean (₹) (SD)	Median (₹) (IQR)	95% CI	
					Lower limit (₹)	Upper limit (₹)
Medical cost						
1	Consultation	0.04%	36.43 (153.27)	0 (0)	13.42	59.42
2	Investigation costs	17.67%	15785.76 (79872.06)	3000 (9400)	3799.42	27772.09
3	Medication	16.86%	15055.85 (39323.29)	4800 (9345)	9154.63	20957.06
4	Hospitalization	46.28%	41340.31 (80799.37)	12000 (31250)	29214.82	53465.81
Total medical cost		80.86%	72218.33 (145815.50)	26000 (45935.50)	50335.77	94100.90
Non-medical costs						
1	Transportation costs	1.14%	1019.10 (2144.73)	300 (865)	1091.59	1732.13
2	Attendant cost or food cost	1.58%	792.77 (1579.96)	200 (1000)	555.67	1029.88
3	Loss of income for patient	13.45%	12010.98 (14006.69)	8000 (12750)	9909.01	14112.96
4	Loss of income for attendant	2.98%	2657.23 (4426.18)	0 (4500)	1992.99	3321.46
Total non-medical cost		19.14%	17099.17 (16245.31)	14100 (16950)	14661.25	19537.09
Total cost		100%	89317.51 (150378.81)	42600 (58730)	66750.28	111884.74

Table 4 presents a logistic regression model that examines the relationship between CHE incidence and various sociodemographic and clinical factors. The model shows that patients in the upper-lower class (p = 0.01), lower-middle class (p = 0.00), and upper-middle class (p = 0.00) are more likely to experience CHE. Also, patients who have had diabetes for five to 10 years (p = 0.00) or more than 10 years (p = 0.03) have a notably higher likelihood of experiencing CHE. Wounds lasting one to three months (p = 0.00) or beyond three months (p = 0.00) had considerably greater risks of CHE than wounds lasting less than one month. An increased likelihood of CHE was observed among patients with deep ulcers (p = 0.00), abscesses with bony involvement (p = 0.00), and localized gangrene (p = 0.03).

**Table 4:** Factors associated with catastrophic healthcare expenditure in patients with diabetic foot ulcers (logistic regression analysis). * Reference category.

Variable	Sub-variable	B	Std. error	Wald	Sig.
Age	Below 40 years	Reference*			
	40 to 60 years	0.64	0.72	0.79	0.38
	Above 60 years	0.94	0.73	1.64	0.20
Gender	Male	Reference*			
	Female	0.171	0.342	0.251	0.62
Occupational status	Unemployed/housewife	Reference*			
	Employed	1.53	0.31	24.99	0.00
Socioeconomic status	Lower class	Reference*			
	Upper-lower class	14.49	0.37	97.01	0.01
	Lower-middle class	14.62	0.39	100.97	0.00
	Upper-middle class	14.89	0.00	130.74	0.00
Comorbidities	Yes	Reference*			
	No	-0.03	0.31	0.01	0.94
Duration of diabetes	Below 5 years	Reference*			
	5 to 10 years	19.48	0.37	1.08	0.00
	More than 10 years	21.14	0.00	4.94	0.03
Duration of wound	Less than 1 month	Reference*			
	1 to 3 months	-1.40	0.38	13.74	0.00
	More than 3 months	-20.12	0.00	15.71	0.00
Wagner’s grading	Superficial ulcer	Reference*			
	Deep ulcer	1.57	0.49	10.18	0.00
	Abscess with bony involvement	1.53	0.49	9.57	0.00
	localized gangrene	1.20	0.52	4.53	0.03
	Extensive gangrene	0.18	0.61	0.09	0.76

Table 5 presents the results of the MANOVA, assessing the impact of various independent sociodemographic and clinical variables on distinct dimensions of HRQoL. The impact of gender on EF (F = 3.27; p < 0.05) and EW (F = 5.69; p < 0.05) was found to be significant. Additionally, occupational status showed a significant association with scores on EF (F = 6.69; p < 0.01) and GH (F = 4.39; p < 0.05). The presence of comorbidities significantly influenced most domains of the SF-36, including PF (F = 7.17; p < 0.05), RE (F = 4.37; p < 0.05), EF (F = 7.70; p < 0.05), EW (F = 17.37; p < 0.001), SF (F = 9.07; p < 0.001), and GH (F = 11.80; p < 0.001). The duration of the wound significantly influenced the PF (F = 8.03; p < 0.01), EF (F = 5.24; p < 0.05), EW (F = 9.52; p < 0.001), BP (F = 13.33; p < 0.001), and GH (F = 6.21; p < 0.01). Wagner's grading of ulcer demonstrated a correlation with diminished scores in PF (F = 3.45; p < 0.05), while CHE significantly influenced majority of domains, including PF (F = 13.45; p < 0.001), EF (F = 8.18; p < 0.001), EW (F = 6.11; p < 0.01), SF (F = 5.32; p < 0.05), BP (F = 5.99; p < 0.05), and GH (F = 12.77; p < 0.001).

**Table 5:** Factors associated with health-related quality of life in patients with diabetic foot ulcers (multivariate analysis of variance). CHE: catastrophic health expenditure; PF: physical functioning; RP: role physical; BP: bodily pain; GH: general health; EF: energy/fatigue; SF: social functioning; RE: role emotional; EW: emotional well-being.

Independent variable	PF (F, p)	RE (F, p)	RP (F, p)	EF (F, p)	EW (F, p)	SF (F, p)	BP (F, p)	GH (F, p)
Age	0.01 (0.92)	0.02 (0.89)	0.04 (0.85)	1.89 (0.17)	2.80 (0.10)	0.73 (0.39)	0.01 (0.92)	0.35 (0.55)
Gender	1.23 (0.27)	0.18 (0.67)	0.77 (0.38)	3.27 (0.04)	5.69 (0.02)	1.57 (0.21)	1.23 (0.27)	3.51 (0.06)
Occupational status	0.23 (0.79)	0.58 (0.56)	0.54 (0.58)	6.69 (0.002)	1.95 (0.14)	0.58 (0.56)	0.49 (0.62)	4.39 (0.02)
Socioeconomic status	0.65 (0.52)	0.06 (0.94)	1.63 (0.20)	1.08 (0.34)	0.90 (0.41)	1.72 (0.18)	0.06 (0.94)	1.06 (0.35)
Comorbidities	7.17 (0.01)	4.37 (0.04)	2.63 (0.07)	7.70 (0.006)	17.37 (<0.001)	9.07 (<0.001)	3.36 (0.05)	11.80 (<0.001)
Duration of diabetes	1.86 (0.18)	0.74 (0.39)	0.54 (0.46)	1.71 (0.19)	0.42 (0.52)	0.98 (0.32)	0.24 (0.62)	0.41 (0.53)
Duration of wound	8.03 (0.005)	0.08 (0.78)	1.02 (0.31)	5.24 (0.02)	9.52 (<0.001)	4.15 (0.04)	13.33 (<0.001)	6.21 (0.01)
Wagner’s grading	3.45 (0.04)	1.51 (0.22)	0.64 (0.53)	0.00 (0.99)	0.01 (0.91)	0.01 (0.98)	2.31 (0.08)	0.19 (0.82)
CHE	13.45 (<0.001)	2.00 (0.16)	0.01 (0.93)	8.18 (0.004)	6.11 (0.01)	5.32 (0.02)	5.99 (0.01)	12.77 (<0.001)

The analysis compares the distributions of HRQoL scores across eight domains among two groups: patients who did not experience CHE and those who did, and the total sample. The total mean scores across domains were as follows: PF (37.23 ± 23.26), RP (17.34 ± 30.55), RE (18.11 ± 32.64), EF (45.06 ± 15.67), EW (52.76 ± 11.74), SF (54.48 ± 17.10), BP (47.49 ± 18.71), and GH (44.83 ± 13.41). In the subgroup analysis, patients who experienced CHE had lower scores in SF-36 domains compared to those without CHE. The mean scores for the CHE group were as follows: PF (39.69 ± 23.30), RP (19.44 ± 32.36), RE (20.58 ± 34.79), EF (46.17 ± 16.09), EW (53.73 ± 12.02), SF (56.79 ± 18.33), BP (48.24 ± 18.80), and GH (47.47 ± 12.90). For participants without CHE, the scores were as follows: PF (35.05 ± 23.14), RP (15.49 ± 28.91), RE (15.94 ± 30.65), EF (44.08 ± 15.32), EW (51.91 ± 11.48), SF (52.45 ± 15.75), BP (46.82 ± 18.70), and GH (42.50 ± 13.50). A statistically significant difference was observed in the general health domain between the groups, with the Mann-Whitney U test yielding a Z score of -2.720 and a p-value of 0.007.

## Discussion

DFU is a severe complication of diabetes mellitus, posing a significant challenge to healthcare systems and society. This issue is particularly concerning in developing countries like India, where limited access to treatments and financial constraints exacerbate disease management. This study examines the economic implications of DFU, emphasizing critical areas of CHE among affected patients. Also, it explores the overall impact on HRQoL, highlighting the urgent need for financial policies and support mechanisms to improve comprehensive DFU management.

The present study was conducted in a self-financed tertiary care teaching hospital, which primarily serves patients from rural regions. The median annual household income of the patients was Rs. 216000 (95% CI: 211336.52-488132.28) ($2743.20 ($2683.98-$6199.28)), with 107 (61.8%) of the population belonging to the middle-class group and 66 (38.2%) belonging to the lower-class category, indicating that a substantial portion of the study population faces economic challenges in accessing healthcare services. The findings of this study are consistent with research by Seshadri et al. [11] and Angadi et al. [17], which also reported that a significant proportion of participants were from lower socioeconomic backgrounds.

A financial analysis of total OOPE revealed an overall amount of Rs. 89317.51 ± 150378.81 ($1135.34 ± 1909.81). Medical expenses accounted for a significant portion, constituting 80.86% (Rs. 72218.33 ± 145815.50) ($917.38 ± 1851.87) of total costs, while non-medical costs represented 19.14% (Rs. 17099.17 ± 16245.31) ($217.16 ± 206.32). Hospitalization was identified as the highest contributor to expenses, further stressing the financial burden imposed by DFU treatment. The variation in healthcare expenditure can be attributed to multiple factors, including inflation rates, differences in healthcare facilities, and disparities in service utilization. Also, indirect costs associated with diabetes, which account for approximately 20% of total costs, highlight the detrimental effect on workforce productivity. Patients with DFU experience significant work absenteeism due to medical conditions and disabilities, adding to the economic burden. Interestingly, this study observes a lower proportion of indirect costs compared to previous research by Seshadri et al. [11] and Angadi et al. [17].

Furthermore, 46.8% of patients in this study reported experiencing CHE, emphasizing the widespread economic strain faced by DFU patients. A study by Seshadri et al. reported that 68.8% of participants had catastrophic expenditures as a result of DFU treatment [11].

In this study, patients with poor socioeconomic status, prolonged diabetes duration, and severe wounds exhibited a significantly higher likelihood of experiencing CHE. Research suggests that ischemia, infection, and prolonged hospitalization increase DFU-related costs, further exacerbating financial hardships [18]. Addressing these factors is crucial for mitigating the risk of CHE in these vulnerable patient populations.

The India Non-Communicable Disease and Injuries (NCDI) Poverty Consortium highlights the critical financial impact of non-communicable diseases on economically disadvantaged populations, particularly in rural India. The substantial costs associated with NCDI not only increase poverty rates but also emphasize the pressing need for targeted healthcare policies aimed at financial risk protection.

The assessment of various sociodemographic and clinical factors reveals significant associations between HRQoL and key variables such as gender, occupational status, wound duration, and Wagner’s grading of ulcers. Specifically, gender was linked to emotional functioning and well-being, occupational status affected emotional functioning and general health, and comorbidities impacted multiple HRQoL domains, including physical and emotional well-being. The study underscores how DFU patients experience substantial limitations in mobility and social interactions, further diminishing their overall QoL. These findings align with a study by Sekhar et al., which reported significantly lower HRQoL among DFU patients compared to non-DFU diabetic patients [19-21]. Similarly, research by Al Ayed et al. in Saudi Arabia found that factors such as education, employment, income, and comorbidities significantly influenced patient health status [19].

The analysis of HRQoL scores between patients with and without CHE revealed that those experiencing CHE generally reported poorer overall health, with statistically significant differences observed in the general health dimension. This suggests that high healthcare costs negatively impact patient well-being, further reinforcing the need for financial interventions to improve HRQoL in affected individuals [22].

Moreover, the increasing costs associated with long-term diabetes management present an escalating challenge for patients and healthcare systems alike [23].

The National Health Policy 2017 aims to increase public health expenditure to 2.5% of the gross domestic product (GDP) by 2030, representing a crucial step toward improving healthcare accessibility and reducing household financial burdens [24]. Furthermore, government initiatives such as the Ayushman Bharat Pradhan Mantri Jan Arogya Yojana (PM-JAY) and Tamil Nadu’s “Medicine at People's Doorstep” (Makkalai Thedi Maruthuvam) scheme provide substantial financial assistance, reducing OOPE for lower-income groups [25]. However, despite these measures, significant gaps remain in protecting DFU patients from financial hardship, necessitating further enhancements in healthcare coverage and affordability.

Limitations

While the study provides valuable insights into the financial burden of DFU care and its impact on HRQoL in India, certain limitations must be acknowledged. The single-center design limits the generalizability of findings to other regions. Furthermore, the cross-sectional nature of the study prevents an assessment of long-term economic impacts, such as post-discharge costs and disability-related expenses. The HRQoL evaluation was based on self-reported measures, which may introduce potential recall bias. To gain a more comprehensive understanding of DFU-related healthcare costs, future multicentric studies incorporating mixed-method approaches and standardized cost-effectiveness models are recommended. Furthermore, longitudinal research examining the prolonged financial effects of CHE on DFU patients could provide deeper insights into healthcare cost burdens and inform policymaking decisions.

## Conclusions

A significant proportion of patients experienced CHE, highlighting the considerable financial impact of DFUs. The study highlights a significant relationship between the economic burden of DFU management and the QoL of individuals affected by the condition. It emphasizes that high healthcare expenditures can lead to financial distress and CHE, adversely impacting patients' well-being. Overall, the findings underscore the need to prioritize both the financial aspects of diabetes care and patient QoL to achieve better health outcomes.
